# Assessing the Attitudes and Perceptions Regarding the Use of Mobile Health Technologies for Living Kidney Donor Follow-Up: Survey Study

**DOI:** 10.2196/11192

**Published:** 2018-10-09

**Authors:** Ann K Eno, Alvin G Thomas, Jessica M Ruck, Sarah E Van Pilsum Rasmussen, Samantha E Halpern, Madeleine M Waldram, Abimereki D Muzaale, Tanjala S Purnell, Allan B Massie, Jacqueline M Garonzik Wang, Krista L Lentine, Dorry L Segev, Macey L Henderson

**Affiliations:** 1 Department of Surgery Johns Hopkins University School of Medicine Baltimore, MD United States; 2 Department of Epidemiology University of North Carolina Chapel Hill, NC United States; 3 Duke University School of Medicine Durham, NC United States; 4 Department of Epidemiology Johns Hopkins School of Public Health Baltimore, MD United States; 5 Department of Health, Behavior, and Society Johns Hopkins School of Public Health Baltimore, MD United States; 6 Center for Abdominal Transplantation Saint Louis University School of Medicine St. Louis, MO United States; 7 Department of Acute and Chronic Care Johns Hopkins School of Nursing Baltimore, MD United States

**Keywords:** follow-up, kidney transplantation, living kidney donor, mobile phone, mHealth

## Abstract

**Background:**

In 2013, the Organ Procurement and Transplantation Network began requiring transplant centers in the United States to collect and report postdonation living kidney donor follow-up data at 6 months, 1 year, and 2 years. Despite this requirement, <50% of transplant centers have been able to collect and report the required data. Previous work identified a number of barriers to living kidney donor follow-up, including logistical and administrative barriers for transplant centers and cost and functional barriers for donors. Novel smartphone-based mobile health (mHealth) technologies might reduce the burden of living kidney donor follow-up for centers and donors. However, the attitudes and perceptions toward the incorporation of mHealth into postdonation care among living kidney donors are unknown. Understanding donor attitudes and perceptions will be vital to the creation of a patient-oriented mHealth system to improve living donor follow-up in the United States.

**Objective:**

The goal of this study was to assess living kidney donor attitudes and perceptions associated with the use of mHealth for follow-up.

**Methods:**

We developed and administered a cross-sectional 14-question survey to 100 living kidney donors at our transplant center. All participants were part of an ongoing longitudinal study of long-term outcomes in living kidney donors. The survey included questions on smartphone use, current health maintenance behaviors, accessibility to health information, and attitudes toward using mHealth for living kidney donor follow-up.

**Results:**

Of the 100 participants surveyed, 94 owned a smartphone (35 Android, 58 iPhone, 1 Blackberry), 37 had accessed their electronic medical record on their smartphone, and 38 had tracked their exercise and physical activity on their smartphone. While 77% (72/93) of participants who owned a smartphone and had asked a medical question in the last year placed the most trust with their doctors, nurses, or other health care professionals regarding answering a health-related question, 52% (48/93) most often accessed health information elsewhere. Overall, 79% (74/94) of smartphone-owning participants perceived accessing living kidney donor information and resources on their smartphone as useful. Additionally, 80% (75/94) perceived completing some living kidney donor follow-up via mHealth as useful. There were no significant differences in median age (60 vs 59 years; *P*=.65), median years since donation (10 vs 12 years; *P*=.45), gender (36/75, 36%, vs 37/75, 37%, male; *P*=.57), or race (70/75, 93%, vs 18/19, 95%, white; *P*=.34) between those who perceived mHealth as useful for living kidney donor follow-up and those who did not, respectively.

**Conclusions:**

Overall, smartphone ownership was high (94/100, 94.0%), and 79% (74/94) of surveyed smartphone-owning donors felt that it would be useful to complete their required follow-up with an mHealth tool, with no significant differences by age, sex, or race. These results suggest that patients would benefit from an mHealth tool to perform living donor follow-up.

## Introduction

Living donation accounted for over a quarter of all kidney transplants performed in the United States in 2016, with over 5500 people serving as living kidney donors [1]. Living donor nephrectomy, while generally safe for properly screened donors, is associated with long-term risks including increased rates of hypertension and end-stage renal disease in specific donor subgroups when compared to similarly healthy nondonors [2-6]. Donor follow-up and engagement are imperative to understand these donation-related risks and improve donor care management and counseling [7,8]. In 2013, the Organ Procurement and Transplantation Network (OPTN) began requiring all US transplant centers to perform living kidney donor follow-up for at least 2 years following live donor nephrectomy. Living kidney donor must complete both a clinical and laboratory component at 6 months, 1 year, and 2 years postnephrectomy. The clinical components include filling out a questionnaire that asks about vital status, working for income, readmission since the last visit, kidney complications, maintenance dialysis, developed hypertension, developed diabetes requiring medication, and cause of death if applicable. Laboratory components include urine protein and serum creatinine [9]. Despite this requirement, living kidney donor follow-up data continues to be missing and incomplete, as reported currently [2,10]. Documented challenges to collecting follow-up information include costs, donor inconvenience, and data collection burden [7,11-13]. The burdens of living kidney donor follow-up might be reduced for both transplant centers and donors by the use of mobile health (mHealth), which is the delivery of health services and resources through mobile devices such as smartphones [14]. As we advance and explore mHealth in solid organ transplantation [15], it is important to understand living kidney donor attitudes, perceptions, and willingness to use mHealth as part of postdonation care management.

Smartphone ownership in the United States increased from 35% in 2011 to 77% in 2016 [16], {, 2017 #9}and smartphones have changed the way patients and providers interact with the health system [17]. Outside of transplantation, mHealth has improved health care access, reduced costs, and increased self-management of chronic diseases in both observational studies and clinical trials [18-25]. Within the transplant community, single-center studies have examined the acceptability and effects of mHealth technologies among organ transplant recipients [26-30]. However, little is known about mHealth attitudes and perceptions among living donors, a distinct population that is generally healthier than the average US adult, without chronic conditions or regular interaction with the health system. After recovering from the donation, living donors may not view follow-up and further engagement with the transplant center as necessary [7].

To understand living kidney donor attitudes and perceptions about using mHealth for follow-up and engagement postdonation, we administered a telephone survey to donors who underwent living donor nephrectomy at our center. The survey included questions on smartphone use, current health maintenance behaviors, accessibility to health information, and attitudes toward using mHealth for postdonation care management.

## Methods

### Survey Design

The survey instrument was developed based on a review of the mHealth literature and pilot tested among clinical transplant providers, living donors, and researchers at our center. The final cross-sectional survey consisted of 14 questions: 4 on smartphone usage, 5 on health maintenance behavior, 3 on accessibility to health information, and 2 on attitudes toward using mHealth for living kidney donor follow-up (Multimedia Appendix 1). The study was reviewed and approved by the Johns Hopkins Medicine Institutional Review Board (NA_00044282).

### Study Population

The study population consisted of 100 living kidney donors who underwent living donor nephrectomy at a large urban transplant center, The Johns Hopkins Hospital in Baltimore, Maryland, and agreed to participate in our study. These donors were part of a 25% (63/252) random sample of living kidney donors at our center (N=252) who had participated in a prospective longitudinal follow-up study, the Wellness and Health Outcomes of LivE Donors (WHOLE-Donor) study, and consented to be contacted for future research (WHOLE study N=1008) [31,32]. We aimed for a sample size of 98 participants based on power calculations to detect a 25% difference between 2 populations (alpha=.05, beta=.80, p1=0.60, minimum sample size N=98). We attempted to contact each individual in our random sample at most 3 times between March and April 2017 and surveyed a convenience sample of the first 100 living kidney donors to agree to the study. The first 100 living kidney donors to agree to participate in the study and provide informed consent took the survey and were classified as participants, leaving the remaining 152 to be classified as nonparticipants.

### Survey Administration

All contact and demographic information for participants was obtained from the WHOLE-Donor study, which is a longitudinal multicenter cohort of living kidney donors. Participants were read an oral consent form, and once consent was obtained, the survey was administered over the phone. Participants were first asked if they owned a smartphone; if they responded no (n=6), the survey was concluded, and no further questions were asked.

### Statistical Analysis

To compare participants who perceived using mHealth for living kidney donor follow-up as useful to those who did not, we separated Likert scale questions into 2 categories. Participants who responded to these survey questions as “slightly useful,” “moderately useful,” or “extremely useful” were categorized as perceiving mHealth technologies as useful. Participants who responded to these questions as “not useful or useless”, “slightly useless”, “moderately useless”, or “extremely useless” were categorized as perceiving mHealth technologies for living kidney donor follow-up as not useful.

We examined associations between participant characteristics and smartphone use and attitudes toward mHealth for living kidney donor follow-up using rank sum for continuous variables and Fisher’s exact tests for categorical variables. All analyses were performed using Stata MP 14.2 for Linux (College Station, Texas, USA).

## Results

### Study Population

From a total of 252 living kidney donors contacted, 39.7% (100/252) participated in the survey, 5.9% (15/252) declined to participate, 7.1% (18/252) had incorrect or no contact information, and the remaining 47.2% (119/252) either did not answer the phone or asked to be called back at another time but were not recontacted before we reached our target sample size.

Of the 100 participants surveyed, 60 were female and 93 were white. The year of kidney donation ranged from 1988 to 2014, with a median (interquartile range [IQR]) of 10 (8-14) years from donation to the time of survey participation. The median (IQR) age of participants was 60 (51-66) years (Table 1). Participants in our study were more likely to be white than potential participants not included in our convenience sample (93/100, 93.0%, vs 126/152, 82.9%; *P*=.03), but there were no significant differences in age, years since donation, or gender of participants and nonparticipants (Table 1).

### Smartphone Usage

Of the 100 participants surveyed, 94 owned a smartphone. All 6 participants who did not own a smartphone were male, 5 were white, and 1 identified as other race. The median (IQR) age of smartphone owners was 59 (range, 50-66) years, compared with the median (IQR) age of 63 (57-68) years for nonsmartphone owners (Table 2). Of the 94 participants who owned a smartphone, 35 owned an Android, 58 owned an iPhone, and 1 owned a Blackberry. Android owners were similar in age and gender but were more likely to be African American individuals (3/35, 9%, vs 1/58, 2%; *P*=.03) compared with iPhone owners, and there were no statistically significant differences in age or gender between Android and iPhone owners (Table 3).

All participants who owned smartphones used them for phone calls, 97% (91/94) for short message service text messaging, 95% (89/94) for internet browsing and accessing apps, 92% (86/94) for email, 67% (63/94) for social media, and 59% (55/94) for video calls. In addition, 32% (30/94) spent <1 hour per day on their smartphone, 51% (48/94) spent 1-3 hours, 13% (12/94) spent 4-6 hours, 3% (3/94) spent 7-10 hours, and 1% (1/94) spent more than 11 hours.

**Table 1:** Study participants’ and nonparticipants’ characteristics.

Characteristics			Participants (n=100)		Nonparticipants (n=152)		*P* value
Age (years), median (interquartile range)			60 (51-66)		58 (50-66)		.37
Years since donation, median (interquartile range)			10 (8-14)		11 (7-15)		.54
**Gender, n (%)**							.52
	Male	40 (40.0)		59 (38.8)		—^a^
	Female	60 (60.0)		93 (61.2)		—
**Race, n (%)**							.03
	White	93 (93.0)		126 (82.8)		—
	Asian or Pacific Islander	2 (2.0)		6 (3.9)		—
	Black	4 (4.0)		17 (11.1)		—
	Other	1 (1.0)		3 (2.0)		—

^a^Not applicable.

**Table 2:** Participant characteristics by smartphone ownership.

Characteristics		Not a smartphone owner (n=6)	Smartphone owner (n=94)
Age (years), median (interquartile range)		63 (57-68)	59 (50-66)
Years since donation, median (interquartile range)		13 (11-20)	10 (8-14)
**Gender, n (%)**			
	Male	6 (100)	34 (36.2)
	Female	0 (0)	60 (64)
**Race, n (%)**			
	White	5 (83)	88 (94)
	Asian or Pacific Islander	0 (0)	2 (2)
	Black	0 (0)	4 (4)
	Other	1 (17)	0 (0)

**Table 3:** Participant characteristics by smartphone type.

Characteristics		Android owner (n=35)	iPhone owner (n=58)	*P* value
Age (years), median (interquartile range)		62 (49-67)	59 (51-63)	.83
**Gender, n (%)**				.65
	Male	11 (31)	22 (38)	—^a^
	Female	24 (69)	37 (62)	—
**Race, n (%)**				.03
	White	30 (86)	57 (98)	—
	Asian or Pacific Islander	2 (6)	0 (0)	—
	Black	3 (9)	1 (2)	—

^a^Not applicable.

### Health Maintenance Behaviors

Of the 94 participants with smartphones, 90 had contacted a health care provider using their smartphone in the past year. All participants who had contacted a health care provider used phone calls. In addition to phone calls, 68% (61/90) used email, 37% (33/90) used short message service text messages, 24% (22/90) used some other medium, and 2% (2/90) used video calls. Of those who used some other medium, 96% (21/22) reported using the internet or apps to connect with a health care provider.

Overall, 85% (80/94) of participants who owned a smartphone were confident that they could maintain a healthy lifestyle, 13% (12/94) were somewhat confident, 1% (1/94) were not confident, and 1% (1/94) reported feeling unsure. In the prior year, 37 participants had accessed their electronic medical record on their smartphone, 38 participants had tracked their exercise and physical activity on their smartphone, and 24 participants had tracked their nutrition, or what they ate or drank, on their smartphone.

### Accessibility to Health Information

In the year prior to study participation, 93 participants had asked a health-related question. Of those, 97% (90/93) had used their doctors, nurses, or other health care professionals to answer their question, 90% (84/93) had used the internet, 74% (69/93) had used friends or word of mouth, 50% (46/93) had used traditional news sources (defined as television, radio, and newspaper), 44% (41/93) had used social media or digital news, 31% (29/93) had used academic or medical journals, and 13% (12/93) had used other sources.

Overall, of participants who owned a smartphone and who had asked a medical question in the prior year, 47% (44/93) most often used their doctors or other health care professionals to answer the question, 38% (35/93) most often used the internet, 9% (8/93) most often used some other resource, 2% (2/93) most often used social media, 2% (2/93) most often used traditional news sources, 1% (1/93) most often used academic or medical journals, and 1% (1/93) most often used friends or word of mouth. When asked to choose the most trusted source of health information, 77% (72/93) reported their doctors, nurses, and other health care professionals; 10% (9/93) reported the internet; 5% (5/93) reported academic or medical journals, 4% (4/93) reported other, 2% (2/93) reported traditional news sources, 1% (1/93) reported friends or word of mouth, and 0% reported social media.

**Table 4:** Participant characteristics by perceived usefulness of smartphone living kidney donor follow-up.

Characteristics		Useful (n=75)	Not useful (n=19)	*P* value
Age, median (interquartile range)		60 (50-65)	59 (50-72)	.65
Years since donation, median (interquartile range)		10 (8-14)	12 (8-12)	.45
**Gender, n (%)**				.57
	Male	27 (36)	7 (37)	—^a^
	Female	48 (64)	12 (63)	—
**Race, n (%)**				.34
	White	70 (93)	18 (95)	—
	Asian or Pacific Islander	1 (1)	1 (5)	—
	Black	4 (5)	0.0	—

^a^Not applicable.

**Table:** 

Characteristics			Useful (n=74)	Not useful (n=20)		*P* value
Age (years), median (interquartile range)			58 (50-64)	63 (53-72)		.03
Years since donation, median (interquartile range)			10 (8-12)	12 (9-17)		.10
**Gender, n (%)**					.43	
	Male	26 (35)		8 (40)	—^a^
	Female	48 (65)		12 (60)	—
**Race, n (%)**					.73	
	White	68 (92)		20 (100)	—
	Asian or Pacific Islander	2 (3)		0 (0)	—
	Black	4 (5)		0 (0)	—

^a^Not applicable.

### Attitudes Toward Mobile-based Follow-up

When asked how useful it would be to complete some of their kidney donor follow-up on their smartphone, 33% (31/94) reported extremely useful, 31% (29/94) reported moderately useful, 16% (15/94) reported slightly useful, 10% (9/94) reported neither useful nor useless, 4% (4/94) reported slightly useless, 2% (2/94) reported moderately useless, and 4% (4/94) reported extremely useless. Those who perceived completing some of their living kidney donor follow-up on their smartphone as extremely, moderately, or slightly useful (75/94, 80%) were similar in age (median 60 vs 59 years, *P*=.65), sex (36/75, 36%, vs 37/75, 37%, male; *P*=.57), race (70/75, 93%, vs 18/19, 95%, white; *P*=.34) and number of years from donation (median 10 vs 12 years; *P*=.45) compared with those who did not find it useful (Table 4).

When asked how useful it would be to access living kidney donor follow-up information and resources on their smartphone, 35% (33/94) reported extremely useful, 31% (29/94) reported moderately useful, 13% (12/94) reported slightly useful, 11% (10/94) reported neither useful nor useless, 5% (5/94) reported slightly useless, 1% (1/94) reported moderately useless, and 4% (4/94) reported extremely useless. Those who perceived accessing living kidney donor follow-up information and resources on their smartphone as extremely, moderately, or slightly useful (79%, 74/94) were younger (median 58 years) than those who did not (median 63 years; *P*=.03) but were similar in sex (26/74, 35%, vs 8/20, 40%, male; *P*=.43), race (68/74, 92%, vs 20/20, 100%, white; *P*=.73), and number of years from donation (median 10 vs 12 years; *P*=.10; Table 5).

## Discussion

### Principal Findings

In this study of living kidney donor perceptions and attitudes toward the use of mHealth for postdonation care management, 79% (74/94) reported that it would be useful to complete some living kidney donor follow-up on their smartphone. This attitude was consistent across age, gender, race, and years since donation. Smartphone ownership was high (94/100, 94.0%), with the majority (58/94, 62%) of participants owning an iPhone. While 77% (72/93) of participants trusted their doctors, nurses, or other health care professionals the most to answer a health-related question, over half (48/93, 51.6%) most often accessed health information elsewhere. These results suggest that an mHealth system for postdonation care management might be welcomed by living kidney donors and also improve donor engagement by facilitating communication between living kidney donors and their transplant center.

Our findings of high interest in mHealth technology among living donors are consistent with prior single-center studies of transplant recipients and candidates. McGillicuddy et al found that 79% of kidney transplant recipients at a single center had a positive attitude toward mHealth for monitoring and managing their medical regimen, and 95% of dialysis patients surveyed at a single center on the kidney transplant waitlist reported interest in using mHealth to increase physical activity [26,27]. Our findings are promising for future engagement of living kidney donors with mHealth technology, despite generally not having prior chronic conditions and being less engaged with the health system.

Additionally, mHealth for living kidney donor follow-up not only has the potential to be useful for donors but also could aid transplant centers in meeting federal data collection and reporting requirements. Currently, more than half of US transplant centers are not able to meet the mandated OPTN policy for living kidney donor follow-up data collection and reporting [10]. Therefore, an mHealth platform that aims to both increase donor engagement and reduce transplant center burden may help with improved follow-up. Moreover, this mHealth system could be reasonably extended to capture survey and other follow-up data beyond 2-years postdonation and provide long-term data on donation-related sequelae.

### Limitations

One limitation of this study is that all participants were from a single center, which may not provide the ability to detect more subtle relationships between attitudes toward mHealth technologies for living kidney donor follow-up and participant demographics. Additionally, our sampling method might have introduced selection bias. However, since this survey was administered as part of a larger ongoing cohort study, we were able to directly compare characteristics between participants and nonparticipants and found that participants in this study were similar to nonparticipants. While our sample size had a low number of African American and other minority individuals, which may have affected our findings, this does reflect national trends of living kidney donation. Finally, the median time since donation of our study participants was 10 years. The majority of these participants donated prior to 2013 when 2-year postdonation follow-up was mandated by the OPTN. Living kidney donors who donated their kidney before the current era of postdonation follow-up data collection and reporting requirements may be less inclined to perceive mHealth for living kidney donor follow-up as necessary; this might have underestimated our already high reported perceived usefulness of mHealth for living kidney donor follow-up in the broader donor population.

### Conclusions

Overall, smartphone ownership in our study was high (94/100, 94.0%), and 79% (74/94) of participants perceived completing some living kidney donor follow-up on their smartphone as useful. These results suggest that patients would be willing to engage with an mHealth system for living kidney donor follow-up and benefit from the implementation of this technology. This work motivates future research to examine the feasibility of implementing such a system in US transplant centers.

## Appendix

Multimedia Appendix 1 Survey questions and possible responses.

## Abbreviations

IQR: interquartile range
mHealth: mobile health
OPTN: Organ Procurement and Transplantation Network
WHOLE: Wellness and Health Outcomes of LivE Donors
