# Supplementary material for: Assessing the Attitudes and Perceptions Regarding the Use of Mobile Health Technologies for Living Kidney Donor Follow-Up: Survey Study
Source: JMIR Mhealth Uhealth. 2018 Oct 9;6(10):e11192. doi: 10.2196/11192 (PMC6231841; doi:10.2196/11192)
Supplement: Multimedia Appendix 1 [file mhealth_v6i10e11192_app1.pdf]

Question	Possible Responses
Do you have a mobile phone that can access the internet?	Yes
	No
What type of mobile "smartphone" do you have? (A "smartphone" today refers to a mobile phone that runs on iOS or Android software).	Android (most google or Samsung devices)
	iOS (most Apple devices)
	Other operating system
	Don't know
I use my phone for: (Mark all that apply)	Phone calls/talking
	Video calls (i.e. FaceTime and Skype)
	Texting
	E-mail
	Social Media
	Internet browsing and using applications (apps)
How much time do you spend on your phone per day doing any of the activities selected above?	Less than 1 hour
	1-3 hours
	4-6 hours
	7-10 hours
	More than 11 hours
In the past year, have you tracked your exercise or physical activity using your phone?	Yes
	No
In the past year, have you tracked your nutrition (i.e. what you eat and drink) using your phone?	Yes
	No
In the past year, please select all the ways in which you have used your phone to connect directly with a health care professional:	Phone calls/talking
	Video calls (i.e. FaceTime and Skype)
	Text-messaging
	E-mail
	Other (please specify)
In the past year, have you accessed your electronic medical record (health records, lab values, etc.) using your phone?	Yes
	No
How confident are you that you can maintain a healthy lifestyle?	Confident
	Somewhat confident
	Not confident
	Unsure
In the past year, what resources have you used to answer a health-related question? (Mark all that apply.)	Your doctors, nurses, or other health professionals (that are not friends or family)
	Academic or medical journals
	Traditional news sources (TV, radio, or newspaper outlets)
	Social media or digital news (Facebook, Twitter, etc.)
	The internet (WebMD, Medline)
	Friends or word of mouth
	Other (please specify)

Of the options you selected above, where do you most often get health information?	Your doctors, nurses, or other health professionals (that are not friends or family)
	Academic or medical journals
	Traditional news sources (TV, radio, or newspaper outlets)
	Social media or digital news (Facebook, Twitter, etc.)
	The internet (WebMD, Medline)
	Friends or word of mouth
	Other (please specify)
Which source of health information do you trust the most?	Your doctors, nurses, or other health professionals (that are not friends or family)
	Academic or medical journals
	Traditional news sources (TV, radio, or newspaper outlets)
	Social media or digital news (Facebook, Twitter, etc.)
	The internet (WebMD, Medline)
	Friends or word of mouth
	Other (please specify)
How useful would it be to complete some of your kidney donor follow-up through your smartphone?	Extremely useful
	Moderately useful
	Slightly useful
	Neither useful nor useless
	Slightly useless
	Moderately useless
	Extremely useless
How useful would it be to access kidney donor follow-up resources and information on your smartphone?	Extremely useful
	Moderately useful
	Slightly useful
	Neither useful nor useless
	Slightly useless
	Moderately useless
	Extremely useless
